# Neotropical Siluriformes as a Model for Insights on Determining Biodiversity of Animal Groups

**DOI:** 10.1371/journal.pone.0132913

**Published:** 2015-07-13

**Authors:** Renata Rúbia Ota, Hugo José Message, Weferson Júnio da Graça, Carla Simone Pavanelli

**Affiliations:** 1 Research Center in Limnology, Ichthyology and Aquaculture (Núcleo de Pesquisas in Limnologia, Ictiologia e Aquicultura - Nupélia), State University of Maringá (Universidade Estadual de Maringá - UEM), Maringá, Paraná, Brasil; 2 Department of Biology, State University of Maringá (Universidade Estadual de Maringá - UEM), Maringá, Paraná, Brasil; University of Girona, SPAIN

## Abstract

We performed an analysis of the descriptions of new species of Neotropical Siluriformes (catfishes) to estimate the number of new species that remain to be described for a complete knowledge on biodiversity of this order, to verify the effectiveness of taxonomic support, and to identify trends and present relevant information for future policies. We conducted a literature review of species descriptions between January 1990 and August 2014. The following metadata were recorded from each article: year of publication, number of species, journal and impact factor, family(s) of the described species, number of authors, age of the authors and coauthors, country of the first author’s institution and ecoregion of the type-locality. From accumulation of descriptions, we built an estimate model for number of species remaining to be described. We found 595 described species in 402 articles. The data demonstrated that there has been an increased understanding of the diversity of Siluriformes over the last 25 years in the Neotropical region, although 35% of the species still remain to be described. The model estimated that with the current trends and incentives, the biodiversity will be known in almost seven decades. We have reinforced the idea that greater joint efforts should be made by society and the scientific community to obtain this knowledge in a shorter period of time through enhanced programs for promoting science, training and the advancement of professionals before undiscovered species become extinct. The model built in this study can be used for similar estimates of other groups of animals.

## Introduction

The discrepancy between the number of species formally described and the number of species that are believed to exist, as well as the lack of knowledge on the actual geographic distribution of species are insufficient, hindering the biodiversity knowledge (see, Linnean [1] and Wallacean [2] shortfalls). Because the understanding of biodiversity is incomplete and evident, it emphasizes the importance of defining the species composition of communities and describing species before their extinction [3].

In an attempt to mitigate these lacks, systematics and taxonomy provide an empirical basis for performing biodiversity inventories, but also for understanding biological evolution and historical biogeographical relationships [4, 5]. However, there are still many difficulties on the progress of taxonomy, known as "the taxonomic impediment" [6, 7, 8]. Mastering the intricacies of morphological and taxonomic descriptions and elucidating the historical biological relationships (phylogenetic and biogeographic) is a task that requires time, specific training and institutional commitment [8]. Additionally, institutions, especially natural history museums that have traditionally employed and trained taxonomists, are now focusing on other projects. However, there are foundations that continue to support descriptive taxonomy, such as the U.S. National Science Foundation in the United States, which is supporting the projects Revisionary Syntheses in Systematics and Planetary Biodiversity Inventory, and the National Council of Scientific and Technological Development (CNPq) in Brazil, which is supporting the Training Taxonomy Program (Protax). The latter and the Partnerships for Enhancing Expertise in Taxonomy in the United States have invested in and trained young taxonomists [8].

Recent analyses (*e*.*g*. [9, 10]) indicated that high rates of new species have been discovered in well-studied regions. The number of publications describing new species has increased in all regions over the last three decades, with proportionately more discoveries in Asia and South America [11]. However, ignoring how many species of each animal group there are still to be described is one of the biggest issues to outline working strategies and future investment in taxonomy.

The fish diversity of Neotropical region is a good model to represent the lack of biodiversity knowledge on a continental scale because it contains the largest number of freshwater fish species [12–14]. Its fish fauna is dominated by Ostariophysi (Characiformes, Siluriformes and Gymnotiformes), which constitute approximately 77% of the species [15]. Among these orders, Siluriformes (catfishes), with just over 3,600 valid species, is one of the most representative in any basin, and of these species, 2,087 are Neotropical [16, 17]. This order was the focus of the All Catfish Species Inventory (ACSI), a project funded by the U.S. National Science Foundation designed to facilitate the discovery, description and dissemination of knowledge on the catfish through a global consortium of ichthyologists and the involvement of dozens of students [18]. Therefore, Siluriformes reflects the behavior of taxonomic data after a major economic and cohesive incentive of researchers involved in the ACSI.

Thus, we have considered the need to review the behavior of the scientific community with regard to its professionals, institutions and journals in the field in relation to such initiatives. We performed an analysis of the new species descriptions of Neotropical Siluriformes both to verify the effectiveness of support for taxonomic research (*e*.*g*. project financing, indexing of specialized journals and training of scholars) and to generate a model that estimates the total number of remaining undescribed species. Additionally, the scope of this work includes identifying current trends and providing relevant information to scientific funding agencies for the development of future policies.

## Materials and Methods

We analyzed some aspects of the species descriptions of Siluriformes between January 1990 and August 2014, due to the main journals began indexing after 1990, and it was also the most productive period in the history of Neotropical ichthyofaunal research [12, 19].

### Data survey

We conducted, between July and August 2014, an extensive literature survey by using the Web of Science (Thomson Reuters), Scientific Electronic Library Online (SciELO), FishBase [19] databases and consulting Reis et al. [20] and Ferraris Jr. [21]. We recorded all the papers in the databases with the word combinations ‘family*’AND‘Siluriformes’ AND ‘Neotropical’, ‘family*’ AND ‘Neotropical’, ‘Siluriformes’ AND ‘Neotropical’, ‘Siluriformes’, and ‘family*’; we recorded all the occurrences of species for ‘family*’ in [20] and [21] and removed the intersections with the databases. The family* represents each family of Siluriformes native to the Neotropical region (Ariidae, Aspredinidae, Astroblepidae, Auchenipteridae, Callichthyidae, Cetopsidae, Diplomystidae, Doradidae, Heptapteridae, Lacantuniidae, Loricariidae, Nematogenyidae, Pimelodidae, Pseudopimelodidae, Scoloplacidae and Trichomycteridae). We only considered the descriptions and taxonomic revisions of valid species according to Eschmeyer [22].

For each paper we recorded: year, number of species described, journal, 2-year impact factor Journal Citation Reports (stratified every 0.3 from 0 to 6.7), families, number of authors, age of authors and co-authors (obtained through the contact with some researchers, catalog cards of master and doctoral thesis or books and, by consulting the *Curriculum Vitae* and social networks), country of origin of first author’s institution and ecoregion of the type-locality according to Abell et al. [23].

### Data analysis

With the dataset obtained in all 25 years period, we described: number of species per year; relative number of species per journal; relative weight (w) of each journal (to identify which journals published more papers describing Siluriformes from the total amount of papers independently of the total number of papers published by each journal, following Braga et al. [24]); Spearman correlation between the percentage of papers per journal and its 2-year impact factor; Spearman correlation between the number of valid and new species described per family; annual mean number of authors and co-authors per article; number of species per age group (every ten years, starting with 20 years old) of authors (production), mean of the number of species per age group by the total number of authors (productivity by author) and mean number of species per age group by the number of authors in the same age group (productivity per age group); number of species per country and per states of the most representative country in the dataset; and number of species by ecoregions.

In the Spearman’s correlation between valid and new species per family, we plotted a theoretical line. Thereby, the points below this line suggest that, in proportion, have high number of studies when compared to the number of species described, *i*. *e*., well sampled families.

### Estimates

We built an accumulation curve of the number of species described annually. By piecewise linear regression with breakpoint fitted by the least-squares method we looked for a change in the trend curve description over the years. We looked for models that best describe the trends described by the curve before and after the breakpoint.

Next, with a binary matrix of species described per year, we estimated the number of undiscovered Siluriformes species in Neotropical region and the time to reaches it. For this, we used two complementary protocols of analysis from community and population ecology. First, we extrapolated the number of species by the second order estimator Jackknife2 (hereafter Jack2) with 1,000 randomizations in *specpool* function in R software [25], Vegan package [26], from community ecology. The *specpool* function is based on the occurrence of species in the sampling sites (in our dataset, each year is analogous to the sampling site) and allows the extrapolation of the richness in a pool of species.

Thereafter, we estimated the remaining time to reach the value ‘Jack2 plus the number of species described before 1990’ in different simulated scenarios with the logistic growth model. We considered each species in our dataset as an individual in a population to extrapolate it through the density-dependent model *Nt* = *K*/[1+(*K*/*N*_*0*_-1]*e* ^*-rt*^), where *K* is carrying capacity, N is number of individuals in the population at time (*t*), *N*_*0*_ is initial number of species in the population and r is a theoretical constant growth rate (for more details, see Gotelli [27]). Then, we considered *K* = Jack2, *N*_*0*_ = 595 (number of species in our dataset). With different values of *r* (0.05, 0.1, 0.2, 0.3, 0.4 and 0.5) we simulated six scenarios (curves) to estimate *t*. Higher *r*-values indicate greater species description rate values. The joint between simulated and real curve that showed a sigmoid was considered the most realistic scenario.

## Results

### Data survey and analyses

**Fig 1:**
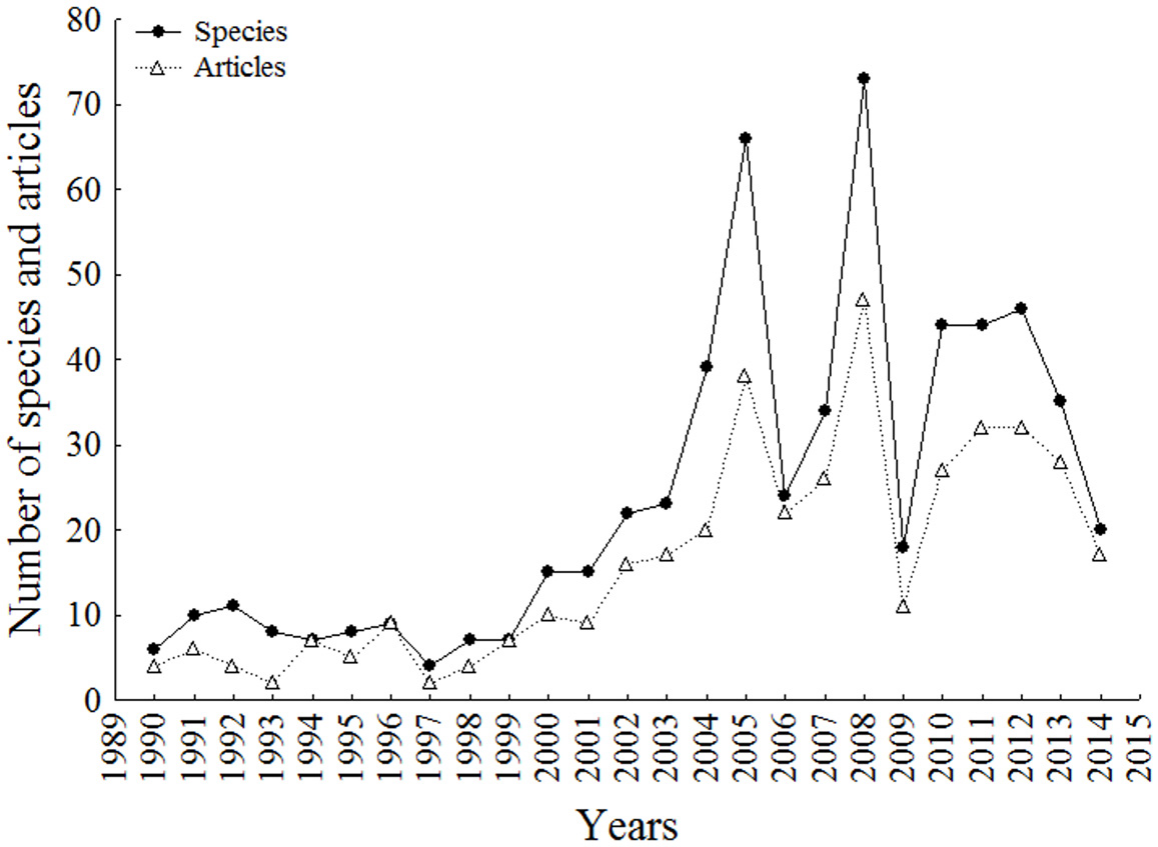
Number of articles and new species of Siluriformes described from the Neotropical region per year. Period analyzed between January 1990 and August 2014.

We found 43 journals, 402 articles, 252 authors and 595 species of freshwater Siluriformes from the Neotropical region described between January 1990 and August 2014. There was a mean of 24 species per year (range 04–73), 1.5 species described per article (range 01–04), and 1.6 articles per author (range 01–05). We observed a marked increase in the bibliographic production primarily between 2003 and 2008 (Fig 1).

**Fig 2:**
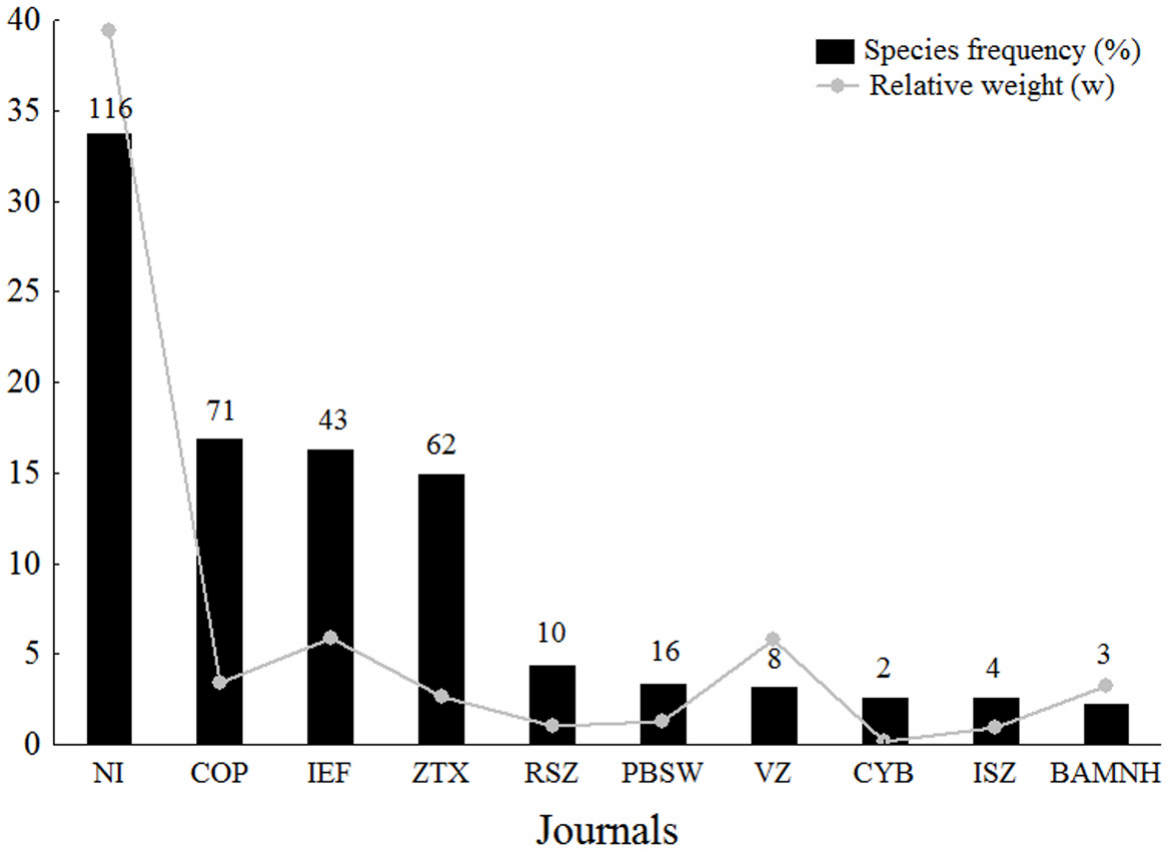
Journals with largest number of new species descriptions. Black bars represent the new species frequency (%) and gray dots represent the given weight of each journal. Numbers above the bars represent the number of papers with new species descriptions for each journal. NI—*Neotropical Ichthyology*; COP—*Copeia*; IEF–*Ichthyological Exploration of Freshwaters*; ZTX—Zootaxa; RSZ–*Revue Suisse de Zoologie*; PBSW–*Proceedings of the Biological Society of Washington*; VZ–*Vertebrate Zoology*; CYB–*Cybium*; ISZ–*Iheringia Serie Zoologia*; BAMNH–*Bulletin of the American Museum of Natural History*.

**Fig 3:**
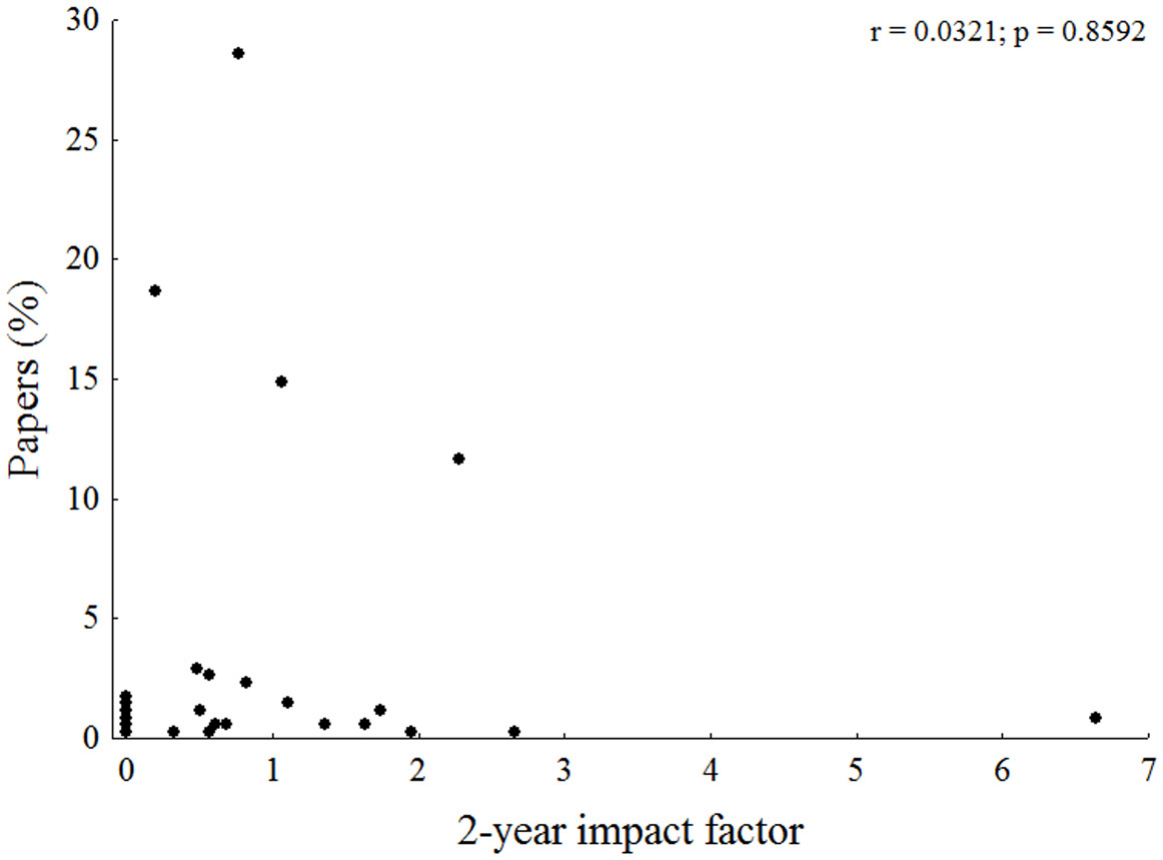
Correlation between the percentages of papers per journal and its respective 2-year impact factor. Dots represents each journal.

Species descriptions were concentrated in the journals *Neotropical Ichthyology* (170 species; 33.7% of frequency), *Copeia* (85; 16.9%) and *Ichthyological Exploration of Freshwaters* (82; 16.3%) (Fig 2). The weight for each journal showed *Neotropical Ichthyology* (39.4), *Ichthyological Exploration of Freshwaters* (5.9) and *Vertebrate Zoology* (5.8) as the most important ones (Fig 2). There was no correlation between the percentage of papers per journal and its respective 2-year impact factor (*r* = 0.03; *p* = 0.85) (Fig 3).

**Fig 4:**
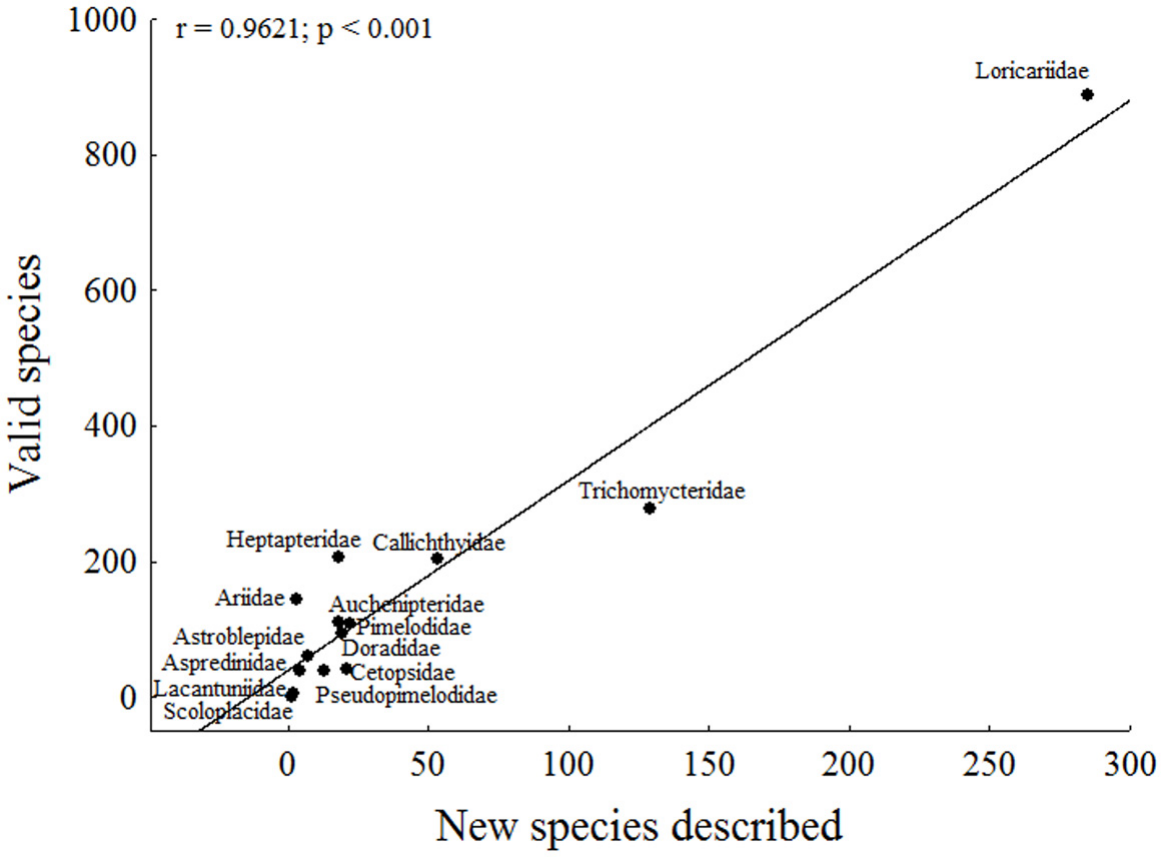
Correlation between the number of valid species and the number of new species described per family. Families well sampled were below the line and low sampled above the line.

The most representative families were Loricariidae (48%, 285 species) and Trichomycteridae (22%, 129). No species described in the analyzed period belonged to Nematogenyidae. The theoretical correlation line showed that the families well sampled (below the line) were: Aspredinidae, Cetopsidae, Lacantuniidae, Pseudopimelodidae, Scoloplacidae and Trichomycteridae; and low sampled (above the line): Ariidae, Astroblepidae, Auchenipetridae, Callichthyidae, Doradidae, Heptapteridae, Loricariidae and Pimelodidae (Fig 4).

**Fig 5:**
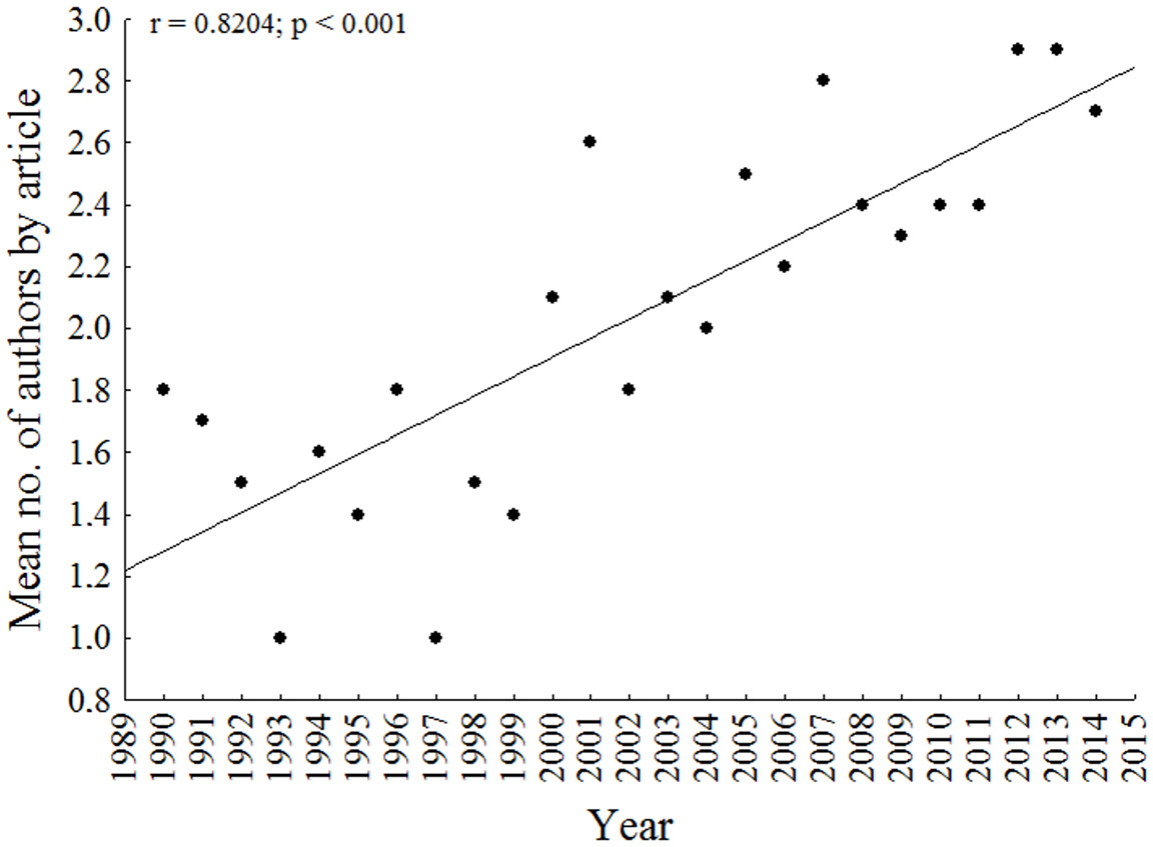
Annual mean number of authors and coauthors per article describing new Siluriformes species. Period analyzed between January 1990 and August 2014.

We observed an increase in the mean number of authors per paper (Fig 5), with the lowest mean (1 author/article) in 1993 and 1997 and highest mean (2.9 authors/article) in 2012.

**Fig 6:**
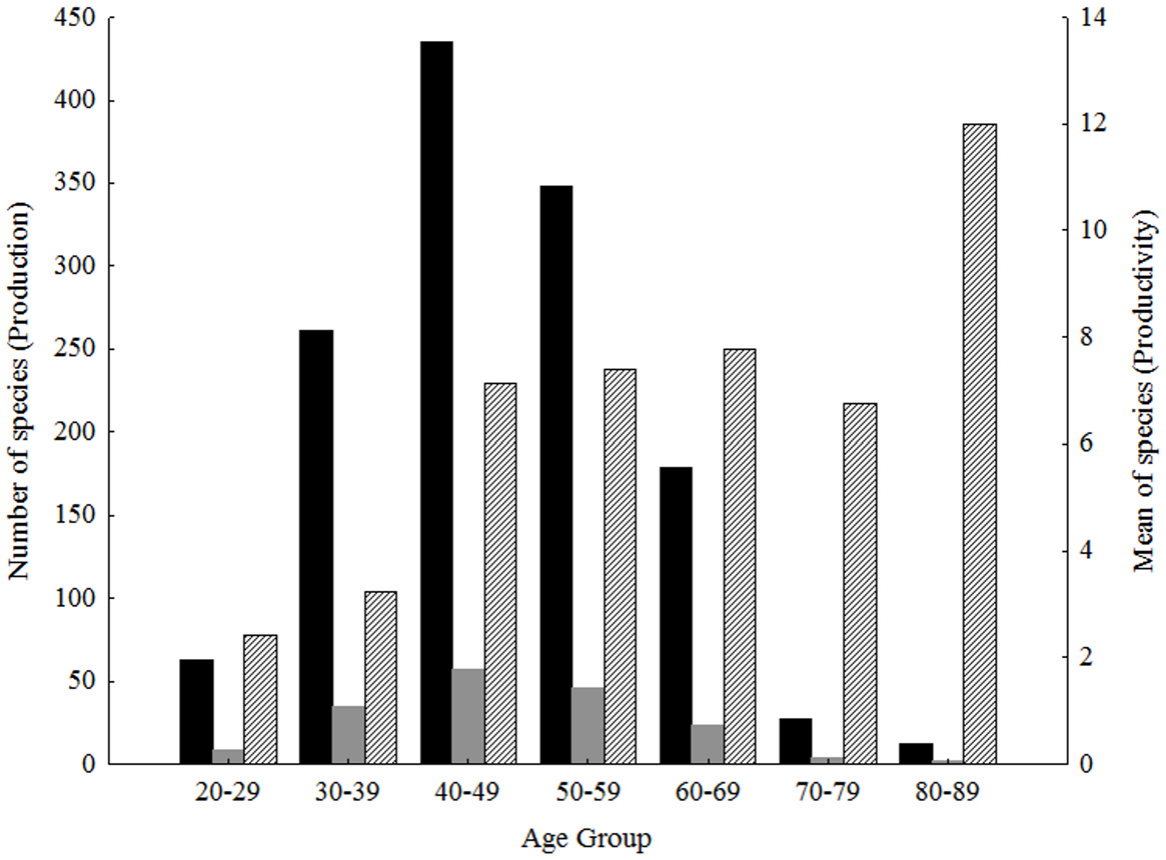
Production, productivity per author and per age group of authors of new Siluriformes species descriptions. Production, black columns, y1 axis; Productivity per author, grey columns, y2 axis; Productivity per age group, striped columns, y2 axis.

The highest production (435 species) related to the age of authors occurred in the 40–49 year age group (Fig 6); this group also had the largest increase in productivity (mean = 1.79), whereas the highest total productivity per age group (mean = 12) was for the group between 80–89 years. Although the productivity of young researchers (20–29 years) was the lowest (mean = 2.42), their productivity by author (mean = 0.26) was not particularly different from that of other age groups (30–39, mean = 1.07; 40–49, mean = 1.79; 50–59, mean = 1.43; 60–69, mean = 0.74; 70–79, mean = 0.11, 80–89 years, mean = 0.05).

**Fig 7:**
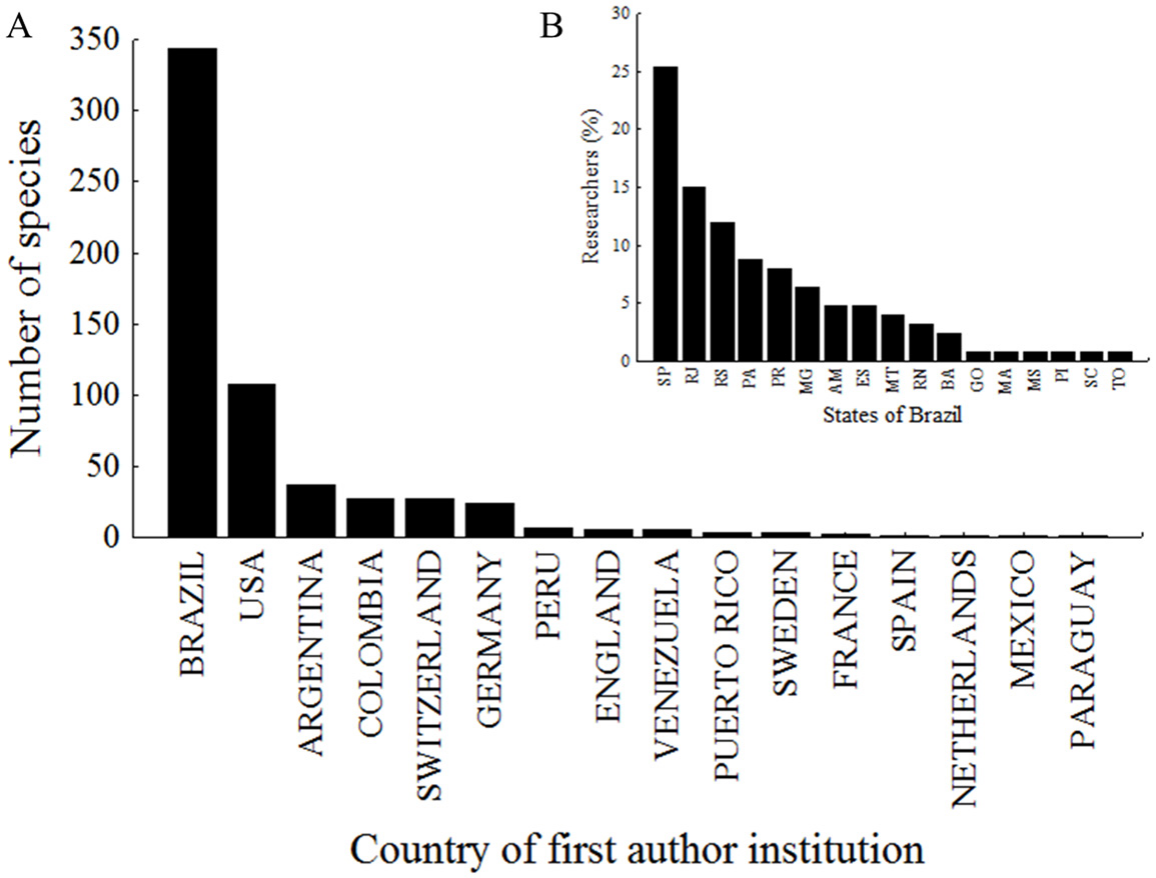
New species described by (A) country of the first author’s institution and (B) percentage of the number of researchers describing new species per state in Brazil. SP—São Paulo; RJ—Rio de Janeiro; RS—Rio Grande do Sul; PA—Pará; PR—Paraná; MG—Minas Gerais; AM—Amazonas; ES—Espírito Santo; MT—Mato Grosso; RN—Rio Grande do Norte; BA—Bahia; GO—Goiás; MA—Maranhão; MS—Mato Grosso do Sul; PI—Piauí; SC—Santa Catarina; TO—Tocantins.

Brazil concentrated the largest number of professionals who described freshwater Siluriformes of the Neotropical region, followed by the United States and Argentina (Fig 7A). In the analysis within Brazil, the species descriptions were conducted in 17 states, concentrated in São Paulo (25%), Rio de Janeiro (15%), Rio Grande do Sul (12%), Pará (9%) and Paraná (9%) (Fig 7B).

**Fig 8:**
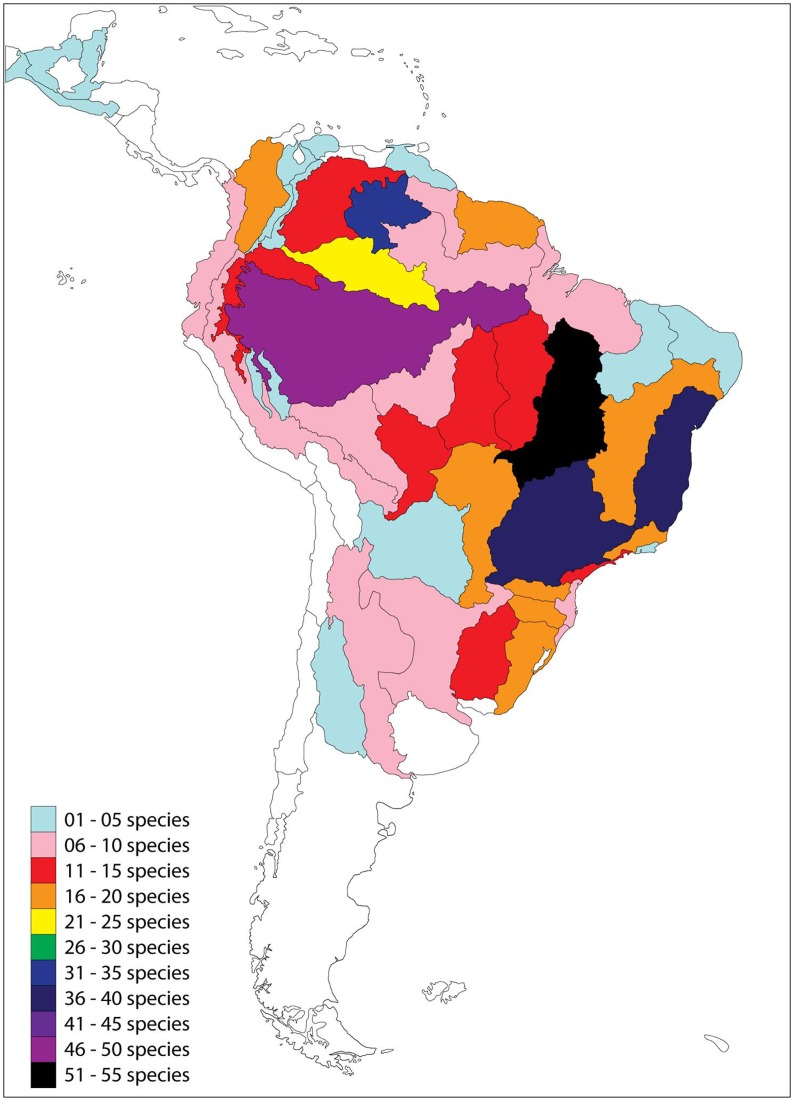
Geographic distribution of new Siluriformes species by ecoregion of the Neotropical region. Ecoregions according to Abell *et al*. [23].

The ecoregions with the most described species were Tocantins-Araguaia (52 species, black colored), Amazonas Lowlands (48 species, purple colored), upper Parana (39 species, dark-blue colored) and Northeastern Mata Atlantica (38 species, dark-blue colored) (Fig 8).

### Estimates

**Fig 9:**
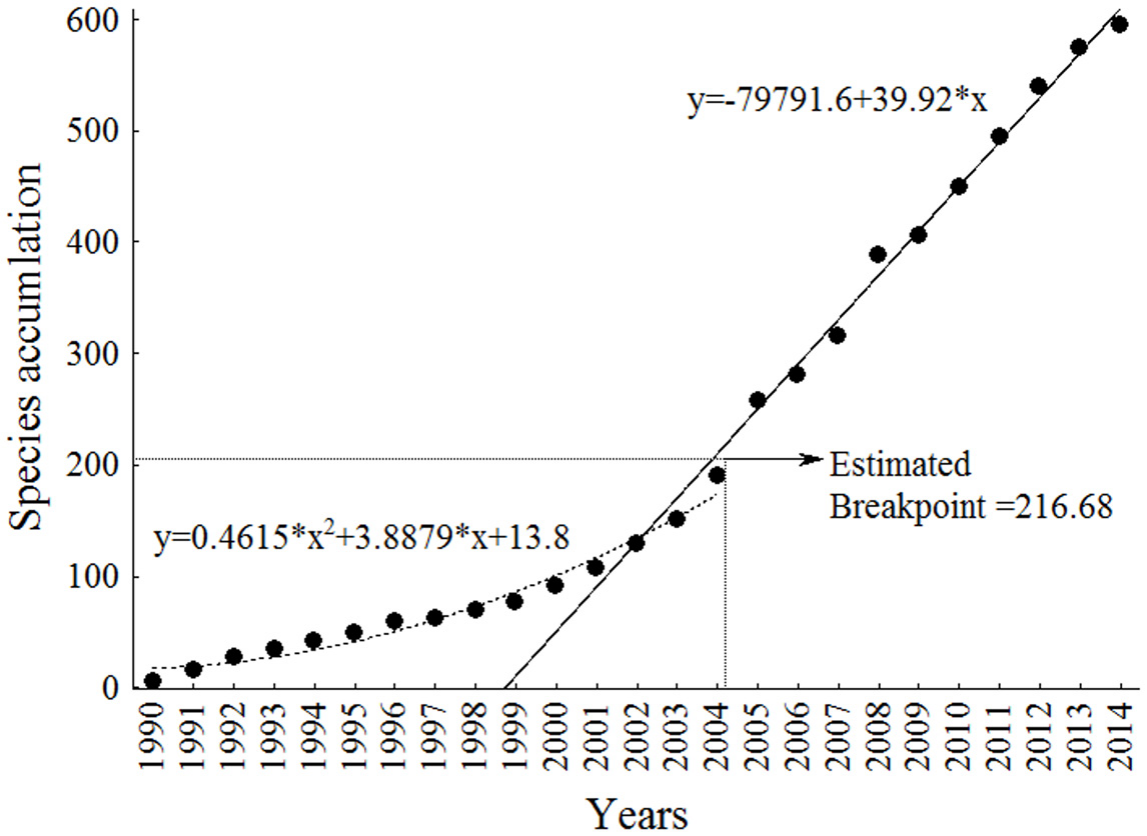
Accumulation curve of the annual production of new Siluriformes species described in the Neotropical region. Curve obtained with piecewise linear regression with breakpoint.

The accumulation curve did not present any trend toward stabilization of species descriptions (Fig 9). The piecewise linear regression estimated the Breakpoint = 217, at the beginning of the year 2004. The curve was described by the quadratic polynomial function *y = 0*.*4615*.*x* ^*2*^ *+3*.*8879*.*x+13*.*8* in the first section (1990–2003) and the linear function *y = -79791*.*6+39*.*92*.*x* in the second section (2004–2014).

Jack2 suggested that, based on our time series, there are 1,715 new species of Siluriformes. Adding 1,452 species discovered before 1990 (prior to our time series), we estimate 3,167 (*K* value) Siluriformes species in the Neotropical region. When we summed 1,452 plus 595 (2.047) species of our dataset, and subtract from 3,167, then we found remaining 1,120 (35%) new species of Siluriformes to be described in the Neotropics.

**Fig 10:**
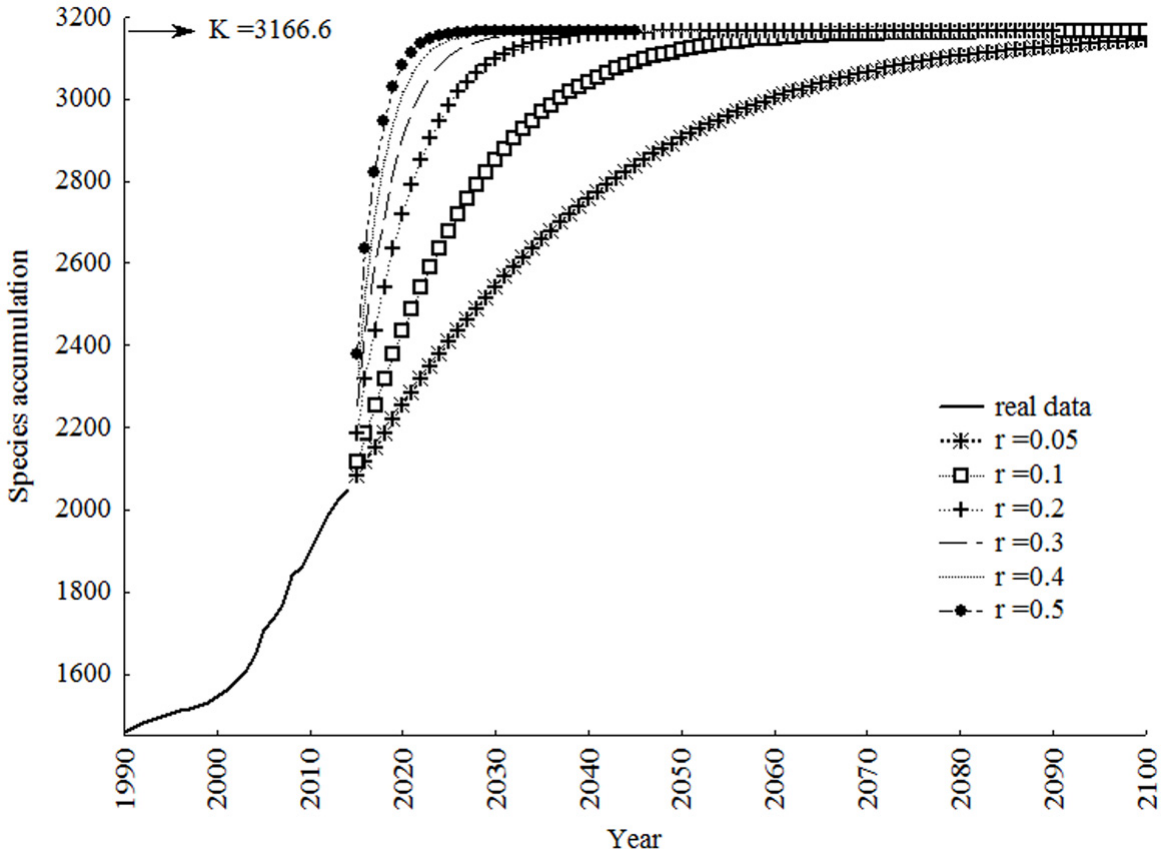
Simulated scenarios with logistic models using different growth rates (*r*). Extrapolation of the accumulation curve of real data until reaching the value estimated by Jackknife2 = *K* = 3,167.

The value *K* = 3,167 used then in the logistic models scenarios suggested that the total number of species will be reached after year 2100 for *r* = 0.05, 2083 for *r* = 0.1, 2049 for *r =* 0.2, 2035 for *r* = 0.3, 2032 for *r* = 0.4 and 2027 for *r* = 0.5 (Fig 10). The model with *r* = 0.1 is the one that changes the overall shape of the curve closer to a sigmoid.

## Discussion

Our data show that the knowledge of Siluriformes diversity has increased in the last twenty five years in the Neotropical region, with an average of 24 new species described per year during the study period compared to 6.3 new species described on average in 232 years since 1758, with the publication of *Systema Naturae* [28], to 1990; although, there are still 35% of species to be described and insufficient knowledge of their geographical distribution. This estimate is similar to that stated by Böhlke et al. [29] for undescribed species of South American freshwater fishes (30 to 40%). The following three factors increased the new species descriptions [22, 30]: division between the Brazilian Meeting of Ichthyology (*Encontro Brasileiro de Ictiologia*) and Brazilian Congress of Zoology (*Congresso Brasileiro de Zoologia*) in 1991, which encouraged the participation of ichthyologists in an unifying and field-focused event; indexing of the journal *Neotropical Ichthyology* (which has the largest number of species described between 1990 and 2014) in 2003 in the Thomson Reuters database; and implementation of ACSI, which was responsible for a 24% increase in the volume of data on Siluriformes diversity over the past 10 years [30]. Additionally, other factors may have contributed to this increment, including increasing in the number of taxonomists of catfishes, intensive exploration of the watercourses of the South America, and changing species concepts that tend to discriminate populations more finely [31].

The concentration of publications in journals with low impact factor showed the restrictive character of taxonomy to explain general features for biodiversity studies. Taxonomic studies are infrequently cited by theoretical managers and monitoring scientists, despite being the most widely used taxonomic metrics for biodiversity quantification. We suggest that all original descriptions of species be fully referenced at least in taxonomic papers and also that taxonomy should be included as part of the training of these scientists to provide a greater familiarity of the morphological, behavioral and evolutionary traits of the species used in studies of populations and communities. Besides, traditionally, developing countries having low financial investment in research [32] and not being English language native [33] act as barriers for publication in high quality journals.

The known diversity in each family and number of researchers working on each of them influenced the number of species described annually and its increase. We also observed a great increase in the mean number of authors per article since 2000, which confirmed the general trend described by Joppa et al. [34], since 1980, and by Langeani et al. [35], since 1990 in the upper Parana River basin. The collaboration among researchers from different institutions has been extremely important to raise the rate of species description.

According to the number of descriptions by young researchers, we observed the increase of taxonomists and the turnover of human resources, due to specific scientific training programs, such as the ACSI project. The most experienced researchers (80–89) showed the highest number of descriptions that indicates the importance of accumulating professional experience.

Although more researchers are involved in species descriptions, their distribution is uneven among Neotropical countries. Most institutions of the first authors are in Brazil (in South and Southeast) United States and Europe, which have surpassed other South American countries. Within Brazil, the largest production is concentrated where there are the major zoological collections and graduate programs [36].

There are different factors that affect the number of species described for an ecoregion. Hydroelectric plants, numerous in Tocantins-Araguaia, for instance, require pre-impoundment impact assessments (EIAs), inventories, monitoring and conservation studies (*e*.*g*. [37,38]); high diversity areas usually have specific initiatives, such as the Fish Diversity of the Principal Channels of the Amazon River, Brazil (Calhamazon) in Amazonas Lowlands; financial effort such as Upper Parana (*e*.*g*. [39–42, 35]); and endemic fish fauna in highly fragmented habitats, such as Northeastern Mata Atlantica (*e*.*g*. [43]). We noted that Fig 8 reflects the number of Siluriformes species described during the analyzed period, but it excludes previously described species as well as species that remain to be described; therefore, it should not be used for conservation purposes.

The first model of the annual accumulation curve exhibits a slight slope and breakpoint between 2004 and 2005, immediately after the first publications of *Neotropical Ichthyology* and its indexing on the Thomson Reuters database as well as after the onset of the ACSI. We believe that the sharped curve after breakpoint has been maintained by the ACSI, whose its samples and training of researchers have caused great reflection in Neotropical ichthyology until nowadays, although it has already been formally finalized.

The Jack2 was plausible with estimative of Böhlke et al. [29] for South American fish in general. Because its theoretically generalist approach, our model can be used for reliable estimates based on data from other animal groups. The curve with *r* = 0.1 was closest to a sigmoid curve. Thus, for this scenario become real, the trend exhibited after 2004 and 2005 must be maintained until the years 2040 and 2050. Only then begin to decline because of its proximity to the carrying capacity (*K* = 3,167). In this case, we predict that in approximately 70 years, the true biodiversity of Neotropical Siluriformes will be known; however, this time period is considered too long when considering the population growth and anthropogenic environment loss. The growth rates *r* = 0.05, *r* = 0.2, *r* = 0.3, *r* = 0.4 and *r* = 0.5 exhibit similar curves, however, these models are dissimilar from a sigmoid curve and does not fit the idealized scenario.

We noticed a steady increase in the descriptions of species as well as a redirection of human and financial resources toward mitigating the lack of that knowledge. Therefore, we also recommend the furtherance of joint efforts by society and the scientific community, programs for scientific training, initiatives such as the ACSI and journals such as *Neotropical Ichthyology*. The training of taxonomists and specific actions that encourage the publication of works describing Siluriformes species are essential to produce the required personnel in a feasible time period before undescribed species have become extinct. The progress of taxonomy should be systematically encouraged with projects such as the ACSI: Calhamazon, Permanent Expedition to the Amazon (EPA), Network of Biotechnology and Biodiversity of the Legal Amazon (Bionorte), PEET, Assembling Tree of Life (AToL), Global Genome Project, Global Taxonomy Initiative (GTI), Conservation, Restoration and Sustainable Use of Biodiversity in the State of São Paulo (Biota-Fapesp), Training program on Taxonomy (PROTAX), Long-term Ecological Research (PELD) and the South American Characiformes Inventory (SACI).

We conclude that our understanding of the actual number of animal species and their actual geographic distribution is far from being fully achieved. In the case of Siluriformes, if the rate of species description remains equal to the trend exhibited after 2004, the 35% of species remain to be described from this order will only be known after 70 years. Other groups of animals that did not have specific initiatives must have even more alarming estimates. The model built here may also be used for similar estimates for them and constitutes a very useful tool to drive strategies and investments to complete the entire knowledge on biodiversity of focused groups or regions.
